# Effect of Anxiety on Empathy: An Observational Study Among Nurses

**DOI:** 10.3390/healthcare8020140

**Published:** 2020-05-21

**Authors:** Diego Ayuso-Murillo, Ana Colomer-Sánchez, Carlos Romero Santiago-Magdalena, Alejandro Lendínez-Mesa, Elvira Benítez De Gracia, Antonio López-Peláez, Iván Herrera-Peco

**Affiliations:** 1 Consejo General de Enfermería, Calle Fuente del Rey, 2, 28023 Madrid, Spain; d.ayuso@consejogeneralenfermeria.org; 2 Faculty of Communication and Art, University of Nebrija, Calle Hostal, s/n, 28240 Madrid, Spain; ana.colomer.universidad@gmail.com; 3 Faculty of Health Sciences, Alfonso X el Sabio University, Avda Universidad, 1, Villanueva de la Cañada, 28691 Madrid, Spain; cromemag@uax.es (C.R.S.-M.); eben@uax.es (E.B.D.G.); 4 Nursing Department, Faculty of Health Sciences, Alfonso X el Sabio University, Avda Universidad, 1, Villanueva de la Cañada, 28691 Madrid, Spain; alendmes@uax.es; 5 Department of Social Work, Universidad Nacional a Distancia, Calle Obispo Trejo, 2, 28040 Madrid, Spain; alopez@der.uned.es

**Keywords:** anxiety, empathy, nursing, personality, 16PF5 questionnaire

## Abstract

Empathy, defined as an emotional ability to put oneself in the place of others, read their state of mind, and think how they are feeling, is an essential pillar of nursing care. On the other hand, anxiety is a frequent emotion that can be generated in stressful work environments, like nurses developing their activity. The aim of the present study is to explore the value of empathy and anxiety personal traits in staff nurses. The sample was comprised of 197 staff nurses from tertiary level hospitals from Madrid, Spain, where 79.2% were women and 20.8% were men in the present study. The instrument used for analysis was the Spanish adaptation of the 16PF5 questionnaire. The results showed the following measurements to warmth (5.58), lively (5.25), socially bold (5.6), privacy (5.82), open to change (5.62), self-reliance (6.12), and anxiety (6.38). Furthermore, anxiety affects positively to warmth (*t*: 2.66; *p* > 0.0001) and lively (*t* = 2.36; *p* < 0.05), but in a negative way to social bold (*t* = −3.17; *p* < 0.001) or open to change (*t* = −5.81; *p* < 0.0001). However, it was not seen to have any effect over privacy (*t* = 1.96; *p* = 0.052) and self-reliance (*t* = 1.19; *p* = 0.234). Finally, it is recommended that healthcare professionals reinforce their personal competencies to strengthen their skills to manage anxiety and improve their empathy competencies.

## 1. Introduction

Currently, health systems globally are operating under multiple social and economic constraints, increased complexity in the working process, and the rapid advance of scientific and technological instruments that are deeply affecting the quality of health professional interaction and communication with patients in clinical settings. In this context, empathy, as well as emerging anxiety disorders, has a substantial impact on patient satisfaction [1,2]. The emphatic ability of nurses is key to quality care, as has been pointed out in nursing research literature since the 1990s.

Empathy is defined as an emotional ability to put oneself in the place of others, read their state of mind, and think how they are feeling [3]; it is considered as a prosocial behavior [4]. It is a multidimensional concept that involves different features like emotion, communication skills, and personal identification with people’s emotions, among others [4,5]. The concept has been evolving over the years, resulting in different theoretical views, all converging to a broad understanding of a dynamic and complex process involving a construct with several components or dimensions [6,7]. Usually is understood that empathy is composed by 3 main dimensions: (i) cognitive (related with knowing what other people are feeling), (ii) emotional (feeling what other people feels), (iii) behavioral (related with ability to communicate the comprehension of perspective from the others) [7,8,9,10]. However, some authors have mentioned the existence of a fourth empathy’s dimension, a moral dimension, related to altruism as a motivation to be empathic towards others [7].

In clinical settings, empathy is an essential personality trait for healthcare professionals that involves professional behavior focused on what the patient and their family expect from the caring staff. Patients’ positives outcomes are directly influenced by the ability of professionals to develop positive relationships to provide quality and safe care and even influence the outcome of illness [7,11,12,13,14,15]. For nurses, as the health care professionals that have the most continued and close contact with patients and their families, empathy is fundamental to ensure good nursing practice, obtain positive patient outcomes, and develop an effective therapeutic relationship with patients [7,16,17,18]. However, it is necessary knowledge worldwide that nurses are aware of the influence of their behavior while caring for their patients [18,19,20,21]. Moreover, evidence has shown that in the healthcare context, especially in the provision of nursing care, the loss or absence of empathy is one leading cause of poor quality and unsafe care [18,19,20,22].

However, nurses usually face intense emotional situations, being mentally assaulted by patients and relatives, witnessing pain, death, and life threatens, among others [23]. These kinds of situations—an excessive personal identification with emotions and feelings of patients—could produce an undesired effect on nurses, namely, the appearance of anxiety and distress and even burnout [5].

Anxiety is a normal emotion, the purpose of which is to trigger individual behaviors and therefore enable the ability of the person to respond. However, when it exceeds in intensity, frequency, or duration, or associated with stimuli that do not represent an actual threat to the human body and produces alterations in the emotional and operational performance, it is considered a pathological manifestation [24]. The anxiety can influence empathic behavior, and this relation has been globally studied in the context of psychology and psychiatry, where it has been observed that anxiety could inhibit a positive relationship with others in their capacity to understand their feelings [25]. Studies have stressed nursing anxiety as a factor of quality of nursing care or how to improve it [12,13,26,27,28]. For instance, the onset of anxiety in health care professionals may result in problems such as imbalances in patient attention and can influence empathy toward the patient and their family, thus affecting professional performance and patient outcomes [12,23].

Anxiety can be seen from different perspectives according to the patient’s needs in different units. Several studies reporting on occupational stress among hospital nurses conclude that the main causes of anxiety are related to working pressure, such as workloads or ethical issues [27,29]. For instance, death and the dying process can be seen in two different stressor perspectives. On the one side, nurses in the oncology and palliative care units would certainly view their patients with compassion, and the anxiety would be expressed as caring for pain and comfort needs, while in the emergency department, anxiety relates to workload, timing, and priorities [30]. Several authors believe that the majority of the stressors prevalent among nursing professionals have to do with external factors, such as lack of suitable working logistics, time pressure, lack of appropriate clinical training, or organizational difficulties [27,29,30,31].

The fact of being anxious can prevent us from appreciating with clarity others and our own behavior, given the negative relationships between empathy and anxiety [32]. Anxiety with egocentrism, highlighting the importance of enhancing empathy because individuals who present high stress have more difficulty in viewing things from other points of view [33,34,35].

It should be highlighted that few studies have focused on these personality traits associated with nurses [36,37,38]. Some authors like Cheung and Yip [39] in a study on depression, anxiety, and symptoms of stress among nurses in Hong Kong considered that lack of studies on these research areas could be due to the generalized idea that to be fit to care, nurses should be “[...] physically and mentally healthy.” It would not be fair and accountable to maintain that nurses are physically and mentally well without evidence [40,41].

The interaction of empathy and anxiety may affect the relationships between health professionals and patients or their families. As the major group providing continuous care to patients in hospitals, nurses experience daily situations that affect their behavior and certainly have an impact on their job performing and patient outcomes [7,36,37,38,39,42,43].

Based on the theoretical proposals above, and have observing a lack of evidence about the analysis between anxiety and empathy, but using the personal traits that define them, the following study was designed to analyze the role of personal traits related with anxiety management and its relation with personality traits related with empathy, in nurses. The aim of the present study is to explore the value of empathy and anxiety personal traits in staff nurses. With regard to this objective, the following research hypotheses are posed:

**Hypotheses** **1.** *Staff nurses are a collective with high personal traits related to empathy*.

**Hypotheses** **2.** *Staff nurses have high values of personal traits related to anxiety management*.

**Hypotheses** **3.** *It is expected that anxiety has more weight in certain components of empathy*.

## 2. Materials and Methods

### 2.1. Participants and Sampling

The questionnaire used in the present study was answered by 203 registered nurses working in tertiary level public hospitals in the Madrid Health Care Service (Spain). Six questionnaires were found incomplete, so the final sample was therefore comprised of 197 participants, from 4 different hospitals of tertiary level: University Hospital of Getafe (58.4%; 115 participants), University Hospital Puerta de Hierro–Majadahonda (29.4%; 58 participants), University Hospital Clínico San Carlos (7.1%; 14 participants), University Hospital Gregorio Marañón (5.1%; 10 participants)

The final sample was comprised of 197 participants with a mean age of 43.16 (SD = 9.52) in a range of 22 to 64 years. Participants distributions by gender was 79.2% (*n* = 156) women and 20.8% (*n* = 41) were men. The mean age of nurses was 43.38 (SD = 9.4) years, where the mean age of men was 39.5 (SD 8.5) years, and the mean age of women was 40.13 (SD 8.7) years.

In relation to work experience, the meantime of work experience as a registered nurse was 20.38 (SD 9.7) years; meanwhile, that meantime of work experience in the current position was 8.42 (SD 6.6) years. The sample and characteristics distribution is shown in Table 1.

The inclusion criteria were (i) nurses on duty and actively working in clinical roles, (ii) nurses developing their activity as staff nurses (without management role), and (iii) nurses with activity in the hospital of tertiary level.

The process of selecting participants was carried out in two well-differentiated steps. In the first step, the main researcher addressed a formal communication about the study to all Nursing Directors of tertiary level hospitals belonging to the MHS, requesting them to inform the nurses and find out their willingness to participate in the research project. In the second step, researchers developed the questionnaires, and it was implemented on a Web platform, which enabled them to be filled in online.

### 2.2. Instrument

*Spanish adaptation of Sixteen Personality Factor Questionnaire: version five (16PF5).* This is an instrument that comprises 185 items organized in 16 scales. 16PF5 analyses the personality factors to appraise and understand personality. It was adapted to the adult Spanish population by Seisdedos [38]. 16PF5 shows an internal consistency for the 16 factors range from 0.54 to 0.84 and acceptable reliability (mean a = 0.75) [43]. Personal traits defined in 16PF5 are primary factors—warmth (A), abstract reasoning (B), emotional stability (C), dominance (E), lively (F), rule-consciousness (G), socially bold (H), sensitivity (I), imaginative (M), privacy (N), apprehension (O), open to change (Q1, self-reliance (Q2), perfectionism (Q3), tension (Q4), suspicious (L); global factors—extroversion (Ext), anxiety (Ax), tough-Minded (TM), independence (In) and self-control (Sc).

The 16PF-5 questionnaire is used to measure both empathy and anxiety. Empathy is characterized by high extraversion and low anxiety. According to the instrument scale, the contributing primary factor to empathy is Warmth (A+), Lively (F+), Socially Bold (H+), Privacy (N−), Self-Reliance (Q2−), and Open to change (Q1+) [42]. On the other hand, anxiety is measure by a direct measure obtains by 16PF5. The mean score to the Spanish population was measure and defined by Seisdedos [38] to personality traits that define empathy. The mean score is defined as 5.5 points; values above this value are expressed with (+), and less than 5.5 are expressed with (−).

### 2.3. Procedure and Data Analysis

This study is quantitative, observational, and cross-sectional.

For data analysis, descriptive and inferential statistics were used via the Statistical Package for the Social Sciences software (SPSS) version 23.0 (IBM, Armonk, NY, USA). First, to identify the relationships between variables, correlational and descriptive analyses were carried out. An analysis of variance was performed for comparison of means, and identification of a possible correlation between anxiety and components of empathy used multiple linear regression. The statistical level of significance set at *p* < 0.05.

### 2.4. Ethical Considerations

The research proposal was previously evaluated and approved by the Ethical Clinical Research Committee of the University Hospital of Getafe of Madrid, Spain, the workplace of the first researcher (reference A11–15). All participants received an invitation letter, additional information about the study, and a request to sign informed consent if volunteering to participate. All participants were assured that both their socio-demographic and personality profile data would be kept under absolute confidentiality, being assessed exclusively by the researchers, according to Spanish legislation. In addition, to ensure the no traceability between compiled information and identity of participants, the questionnaires were only identified with a code as a reference to introduce all the information in the database. Data were collected and analyzed between August and November of 2018.

## 3. Results

### 3.1. Assessment of the Personality Traits that Define Empathy and Anxiety

The analysis of the personality traits related to empathy in the group of participants (*n* = 197) shows 6 items above the mean score (5.5) to the Spanish population. The traits above the mean value were: warmth (5.58), socially bold (5.6), privacy (5.82), open to change (5.62), and self-reliance (6.12). Related to anxiety, we can observe a mean value of 6.38 above the mean value to the Spanish population too (Table 2).

In order to analyze the possible effects of variables like sex, age, experience as nurse or experience in current position over anxiety or components of empathy, we observed the existence of a correlation between sex and warmth and age and lively, but no correlation between the remaining variables and empathy components.

As shown in Table 3, exists a positive correlation between sex (women) (*r* = 0.6; *p* < 0.0001; 95% CI (1.26; 1.66)) and warmth.

### 3.2. Descriptive and Correlational Analyses of Anxiety and Empathy

Table 4 shows the relationships observed between all the components of empathy and anxiety in the complete sample of nurses. We can observe that warmth (*t*: 2.66; *p* > 0.0001) and lively (*t* = 2.36; *p* < 0.05) was correlated positively with anxiety, but there was a negative correlation between anxiety and socially bold (*t* = −3.17; *p* < 0.001) or open to change (*t* = −5.81; *p* < 0.0001).

On the other hand, we can not observe any relation between anxiety and privacy (*t* = 1.96; *p* = 0.052) and self-reliance (*t* = 1.19; *p* = 0.234).

## 4. Discussion

The empirical study presented above met the objective originally posed, to determine the relational role of anxiety over the different empathy components.

In the first place, and in relation to personal trait measures related to empathy, the results showed that the nurses were above the mean score (5.5 points) to Spanish population. Staff nurses included in our study were adjusted to values defined to be considered an empathic person: warmth (+), lively (+), privacy (−), open to change (+) and self-reliance (−) [38,42,44]. These data suggest that staff nurses are affable, cooperative and that enjoys the company of others and receptive to other points of view and opinions, characteristics that have a very specific bearing on the role of nurses over the patients’ care [44]. This result is according to previous studies which have identify nurses as empathic people [16,31]. These findings coincide with our first research hypothesis in which staff nurses are a collective with a high personal traits related with empathy. The situation differs from studies that have shown that the nurse–patient relationship is poor and nurses rarely spend time to speak and empathize with patients [20,21]. From a global perspective, nurses acknowledge that empathy, professional competency, and the use of adequate communication are important qualities nurses should have to establish a therapeutic relationship or appropriate caring support [22].

An important result obtained in this study is the high level of anxiety that has been detected: 6.38 points while the mean measure in Spanish population is 5.5 points [34]. This result is in accordance with other research showing that the prevalence of anxiety in nurses is higher than in the whole population, indicating that registered staff nurses as a collective come under different kinds of pressure related with their clinical activity [27,29,31] that finally predispose to mildly alterations like anxiety [22,40,41]. These findings coincide with our second research hypothesis in which staff nurses are a collective with a high values of personal traits related with anxiety management.

In the second place, the results obtained in our study showed that there were no statistically significant differences between sex, age, experience in actual position or even experience as a nurse with anxiety or empathy’s components. As a exception to the previous data, we observed the existence of a relationship between sex, women, and warmth, which could be associated with the auto-perception based on gender bias about the role of caregivers, nurses in particular. Nurses are a feminized collective; in Spain, women represent 84.2% of total nurse workforce [45], are associated with high warmth, a characteristic associated to women, and nurses are a collective constituted mostly by women [46]. This may be caused by several reasons, including selection bias due to nursing being a feminized profession.

In the third place, advancing in the study, the objective of analyzing the effect of anxiety over empathy’s components was met. The findings showed that anxiety affects the components of warmth, lively, social bold, and open to change; however, it was not seen any effect over privacy and self-reliance. We observed a positive relationship between anxiety and warmth and lively, which means that when anxiety levels rise, nurses could increase their interest in meeting better people and need more attention from others [47]. Empathy begins when listening to someone, without preconceived assumptions, with the only purpose of providing help or support. This effort is carried out not only emotionally but also within a semiological level because the empathy must be comprehensive and involves the understanding of psychological, physical, and social suffering. These findings coincide with our third research hypothesis, in which it was expected a priori to find that anxiety has more weight in certain components of empathy.

On the other hand, our results also emphasize that anxiety could negatively affect the components of social bold and open to change. Healthcare professionals and nurses, in particular, in high anxiety situations tend to not be bold people and may even become a withdrawn person [48,49]. So this anxiety would induce changes in nurses that are derived from physical and psychological exhaustion [50,51]; nurses tend to be less adaptable to changes in their work environment and even less susceptible to patient needs.

Finally, our data showed that privacy and self-reliance are empathy competences totally independent from anxiety. In relation to privacy, we observed that anxiety does not influence the nurses’ concept of privacy. This could be explained because privacy is a complex concept, defined by four elements: (i) solitude, (ii) reserve, (iii) intimacy, and (iv) anonymity [52], and some of them are impossible to apply correctly in the nursing work environment, where they are more focused on privacy from a physical dimension but not from a psychosocial dimension [53]. About self-reliance, it is a competence associated with the self-confidence in your own ability to make decisions [54]. Our data showed us that self-reliance was independent of anxiety, an opposite result previously described where anxiety negatively affects decision-making [55]. A possible explanation to this discrepancy between results could be based in the ability on decision-making are highly influenced by experience in clinical practice [56], as we observed in our study, the mean experience time as registered nurses (obtained clinical practice) was 20.38 (SD 9.7) years, data that showed a sample with clinical practice and experience. On the other hand, experience was associated with self-confidence, a skill that influence decision-making, but was also associated with protocols and teamwork in nurses [56]. Nurses have high competences in identifying patient situations and patterns [55]) that allow them to recognize and apply the standard protocol in nursing practice, with the aim to offer the best care to patients.

Therefore, organization policies need to promote not only management and leadership competencies among nurses but also specific training skills to deal, understand, and perceive signs and symptoms of anxiety, having in mind that anxiety is associated with burnout in the work environment [40,56]. Similarly, the development and implementation of continuous and personal development educational methods to reinforcement empathy, like role-play or simulation games, are also essential as a basic competence among nurses [31]. The implementation of these policies helps to ensure the best physical and psychological conditions for nurses, facilitating the well-being of nurses inside and outside the work environment.

This study has some limitations. First of all, a limitation is related to study design because it is an observational and cross-sectional study, where no causal relationship can be established between variables. Secondly, our study is based on self-report measures used to evaluate personal traits related to empathy and anxiety.

## 5. Conclusions

The findings exposed in the present study emphasize the role of anxiety, and its relationship with the different components of empathy. Our results suggest that staff nurses are affable, cooperative, enjoy the company of others, and are receptive to other points of view and opinions, characteristics that have a very specific bearing on the role of nurses over patient care. However, a high level of anxiety is also related with changes that are derived from physical and psychological exhaustion, making staff nurses less adaptable to changes in their work environment and even less susceptible to patient needs. Finally, anxiety does not influence the nurses’ concept of privacy and their own ability to make decisions (based on their clinical experience).

The present study represents an advance in the knowledge of this association, exploring the ways by which anxiety and empathy are related in nursing. Its practical implications would enable the development of psychological competencies and tools for nurses, not only for registered nurses, so it would be advisable for nursing students to receive training in the personal competencies.

Based on these findings, it is recommended to develop strategies to monitoring nurses’ anxiety levels in their clinical activity but to check empathy competencies, related to their relationship with other healthcare professionals and the relationship with the care to patients. Empathy is one of the most important elements in care, not only because is necessary to attend empathically to patient needs or requirements, but is also important as a way to establish a good relationship with other healthcare professionals and strengthened the work team.

The development and implementation of training programs focused in strengthened personal competencies related with empathy, like communication skills or emotional competencies, among others, is recommended to health institutions administrators and policymakers to enable the safe environment and quality working conditions. These strategies and actions can be improved through the implementation of training programs and interventions to increase empathy and reduce anxiety in nurses.

## Figures and Tables

**Table 1:** Distribution and sample characteristics.

Variables	*n*	%
Sex		
Female	156	79.2
Male	41	20.8
Age (years)		
<40	86	43.66
>40	111	56.34
Experience in actual position (years)		
<8	98	49.74
>8	99	50.26
Experience as a nurse (years)		
<20	103	52.28
>20	94	47.72

**Table 2:** Personality factors for empathy and anxiety.

CAS Traits	Mean	SD
Warmth	5.58	1.62
Lively	5.25	1.78
Socially bold	5.6	1.74
Privacy	5.82	1.92
Open to change	5.62	1.4
Self-reliance	6.12	1.51
Ax	6.38	1.85

**Table 3:** Relationship between socio-demographic variables, empathy components, and anxiety.

	Sex	Age	Exp. Act. Pos.	Exp. Nur.
	*F* (Significance)			
Warmth	6.5 (0.012) *	0.99(0.496)	1.27(0.19)	0.69(0.916)
Lively	0.02 (0.89)	0.11 (0.21)	0.84(0.694)	1.37(0.089)
Social Bold	0.04 (0.84)	1.03(0.433)	1.02(0.443)	1.58 (0.25)
Privacy	0.57(0.452)	1.28(0.144)	0.92(0.578)	0.71(0.895)
Open to change	0.04 (0.847)	0.9 (0.64)	0.63(0.918)	0.88(0.676)
Self Reliance	0.19 (0.66)	1.27(0.155)	0.81(0.731)	0.62(0.961)
Anxiety	2.79 (0.096)	0.93(0.588)	0.69(0.865)	0.86(0.71)

Exp. Act. Post means experience in actual position (in years); Exp. Nur. means experience as nurse (in years); * means *p* < 0.05.

**Table 4:** Correlation pairs to anxiety and empathy items.

Pairs		*T*	Significance	95% CI	
				Lower	Upper
Ax	Warmth	2.66	***	0.06	0.39
	Lively	2.36	*	0.03	0.34
	Socially bold	−3.17	**	−0.38	−0.09
	Privacy	1.96	n.s. (0.052)	0.04	0.3
	Open to change	−5.81	***	−0,62	−0.3
	Self reliance	1.19	n.s. (0.234)	−0.07	0.03

n.s. means no significance; * means *p* < 0.05; ** means *p* < 0.001; *** means *p* < 0.0001.
